# Cortical high gamma network oscillations and connectivity: a translational index for antipsychotics to normalize aberrant neurophysiological activity

**DOI:** 10.1038/s41398-017-0002-9

**Published:** 2017-12-18

**Authors:** A. Ahnaou, H. Huysmans, T. Van de Casteele, W. H. I. M. Drinkenburg

**Affiliations:** Department of Neuroscience, Janssen Research and Development, a Division of Janssen Pharmaceutica NV., Turnhoutseweg 30, Beerse, 2340 Belgium

## Abstract

Oscillatory activity in the gamma frequency range is a critical mechanism, which integrates neural networks within and across brain structures during cognitive processes. In schizophrenia, abnormalities in high gamma oscillations are ubiquitous and most likely reflect dysfunction in neuronal networks. In conscious rats, disturbed network oscillations associated with positive symptoms and cognitive deficits were modeled in different cortical areas by the dopaminergic agonist (amphetamine) and the N-methyl-D-aspartate (NMDA) receptor antagonists (PCP and MK801). Subsequently, the efficacies of marketed atypical antipsychotics (olanzapine, risperidone, and clozapine) to normalize dysfunctional oscillations and network connectivity were examined. Acute NMDA antagonists elicited aberrant synchrony in the gamma frequency oscillations. In addition, coherent slow alpha network activity was observed with MK801 and amphetamine, both of whose oscillatory rhythms were correlated with pronounced locomotor activity. All antipsychotics commonly decreased slow alpha and high gamma network oscillations in different cortical regions as well as motion behavior. In the combined treatments, antipsychotics attenuated NMDA antagonist-induced abnormalities in functional network oscillations and connectivity, whose effects on motor behavior is mechanistically related. These results suggest that pharmacologically induced disruption of cortical gamma oscillations and network connectivity in rats is a candidate model to study dysfunctional oscillatory patterns described in positive and negative symptoms of schizophrenia. The efficacy of antipsychotics to rescue cortical network oscillatory patterns is in line with the idea that glutamatergic and dopaminergic systems play a role in maintaining the integrity of cortical circuits. Thus, gamma oscillations could provide a powerful translational index to assess the integrity of neural networks and to evaluate the efficacy of drugs with potential antipsychotic properties.

## Introduction

Ongoing brain oscillations determine the dynamic changes in brain states, and influence alertness such as cortical computations, cognitive perceptual grouping, attention-dependent stimulus selection, subsystem integration, working memory, and consciousness^1–10^. Temporal oscillation on alpha rhythm reflects an active inhibitory mechanism of task-irrelevant information, whereas gamma rhythm is critical for the maintenance of working memory^11–13^.

Network oscillations have received much interest in contemporary schizophrenia research as the same cognitive processes driven by gamma rhythm are disrupted in this disorder^14,15^. Alteration in GABA-mediated neurotransmission has been proposed as a candidate mechanism that impairs gamma oscillations^16–21^. Postmortem studies in schizophrenics confirmed deficits in GABA-mediated synaptic transmission and reduced GABA synthesis in the parvalbumin (PV) containing subpopulation of inhibitory neurons^22^. PV interneurons are crucial in the genesis of gamma oscillations in cortical circuits, as they exert powerful, precisely timed recurrent inhibition onto their target pyramidal cells and inhibitory interneurons^16,23,24^. These GABAergic interneurons appear to be under the control of glutamatergic system, which is also known to be abnormal in schizophrenia^18,25–29^.

Electrophysiological findings have firmly established the role of abnormal oscillatory processes in schizophrenia^5, 30^. Gamma oscillatory rhythm can be assessed across species and at various spatial levels, from single unit to large-scale networks' electroencephalographic (EEG) recordings, which offers the possibility of applying findings from basic neuroscience models to clinical studies. Activation of dopaminergic receptors by amphetamine or blockade of N-methyl-d-aspartatic acid (NMDA) receptor by ketamine, PCP, and MK801 has widely been used in humans, primate, and rodents to recreate core symptoms of schizophrenia, such as hallucinations, thought disorder, negative symptoms, and cognitive deficits^31–36^. Previous reports showed aberrant increases of gamma frequency oscillations in acute psychotic unmedicated schizophrenic patients, and activation of dopaminergic receptors or blockade of NMDA receptors resulted in a short-term pathological increases of gamma oscillations in local cortical circuits in humans and animals^37–42^. Thus, network gamma oscillatory rhythm may provide a valuable window for investigating the contribution of dopaminergic and glutamatergic transmission in the disturbance of integrative circuitry of cognitive processing and could possibly help to improve detection of more effective and targeted novel pharmacological therapies^43–45^.

The present studies aimed to evaluate whether EEG network oscillations in conscious rats are candidate quantitative markers to pharmacologically recreate cardinal features of dysfunctional cortical networks described in schizophrenia, and to subsequently assess the efficacy of antipsychotic drugs to normalize aberrant functional network activities and associated disorganized motion behavior.

## Materials and methods

### Animals and surgical procedure

All procedures were performed in accordance with the guidelines of the Association for Assessment and Accreditation of Laboratory Animal Care, and of the European Communities Council Directive of 24 November 1986 (86/609/EEC) and were approved by the Janssen Pharmaceutica ethical committee. The experiments were carried out in male adult Sprague–Dawley rats, supplied by Harlan (the Netherlands), and weighing ~250 g at the time of surgery. Animals were housed in full-view Plexiglas cages (25 × 33 cm, 18 cm high) that belong to IVC racks (individually ventilated cages) located in a sound-attenuated chamber. Rats received a chip for identification purpose by Animal Inventory and Weighing system, and were maintained under controlled environmental conditions throughout the study: 22 ± 2 °C ambient temperature, the relative humidity at 60%, 12:12 light–dark cycle (lights off from 06:59 a.m. to 18:59 p.m., light intensity: ~100 lux) and food and water available ad libitum.

Surgery was performed under Isoflurane anesthesia as described earlier (Ahnaou et al.^46^ In brief, animals were equipped with six stainless steel-fixing screws (diameter 1 mm) for the recording of EEG activities inserted bilaterally in the left and right hemispheres along the anteroposterior axes at the locations (frontal left “FL”, parietal left “PL”, occipital left “OL”, and frontal right “FR”, parietal right “PR”, occipital right “OR”, respectively). All electrodes were placed stereotaxically (FL/FR: AP + 2 mm, L ± 2 mm; PL/PR: AP −2 mm, L ± 2 mm and OL/OR: AP −6 mm, L ± 2 mm from Bregma). In addition, stainless steel wires (7N51465T5TLT, 51/46 Teflon Bilaney, Germany) were placed in the muscle of the neck to record the electromyogram activity (EMG). Electrodes were connected to a pin (Future Electronics: 0672-2-15-15-30-27-10-0) with a small insert (track pins; Dataflex: TRP-1558-0000) and were fit into a 10-hole connector, after which the whole assembly was fixed with dental cement to the cranium.

### Recording, analysis of spectral power, and network connectivity

Two weeks after recovery, all EEG recordings from six brain regions were performed under vigilance-waking condition during the dark circadian phase as described earlier^46^. In addition to EMG activity, general motor level was monitored in the home cage by two passive infrared detectors placed above each recording cage, and the envelope activity was used to analyze motion levels following drug administration. Artifact-free waking epochs with low-voltage fast EEG activity, high to moderate EMG, and body activities were considered in the analysis. Epochs with high-voltage slow cortical waves in the absence of EMG and locomotor activities were discarded. A notch Finite Impulse Response (FIR) filter at 50 Hz was applied to avoid voltage related to power line interferences.

All experimental baseline EEG recording sessions of 30 min started 2 h after the light offset to avoid confounding circadian effect on EEG. In case of dose–response experiments, signals were recorded for 2 h after vehicle or drug administration (*n* = 8 for each condition). For reversal challenge experiments, antipsychotics or vehicle were administered, followed 30 min later by a challenge drug and EEGs were further recorded for 2 h (*n* = 8 for each condition). Continuous EEG and EMG field potentials were acquired at 2 kHz sample rate with an input range of ±500 mV through a Biosemi ActiveTwo system (Biosemi, Amsterdam, the Netherlands), which replaces the conventional ground electrodes by two separate electrodes: the common mode sense active electrode and the driven right leg passive electrode. This common mode reference for online data acquisition and impedance measures is a feedback loop driving the average potential across the montage close to the amplifier zero. In addition to motion levels, the EMG signals were used to differentiate active vs. passive behavior. The signals were amplified and analog band-pass-filtered between 1 and 100 Hz and was digitized with 24-bit resolution.

Analysis was performed using a method described earlier^46^. In brief, spectral density estimates were calculated using Fast Fourier Transform with Hanning window function; block size of 512 data points, giving 1.0 Hz resolution, and power was expressed as percentage of total power over 1–100 Hz. The average spectral density in each frequency oscillation was normalized across animals to obtain the full power spectrum (heat map with 1 Hz resolution) constructed from overlapping windows of 4 s data and plotted for post-drug periods windowing over 1–100 Hz. Each frequency band was averaged for each 15-min interval in the following frequency bands: Delta band (1–4 Hz), Theta band (theta1: 4–6.5 Hz, theta2: 6.5–8 Hz), Alpha band (alpha1: 8–11 Hz; alpha2: 11–14 Hz), Beta band (beta1: 14–18 Hz; beta2: 18–32 Hz), and Gamma band (gamma1: 32–48; gamma2: 52–100 Hz).

In each experiment, values of baseline 4 s waking epochs were averaged for 30 min followed by 15 min average for the remaining time after the pharmacological treatments. Drug-induced changes in different cortical networks and EEG power were calculated for each 15 min time block over 2 h as the ratio of mean spectral power obtained following the administration of test drug vs. the mean spectral power obtained during 30 min baseline period. This procedure allows for assessment of drug-induced changes in each frequency band as a percentage of the original power, compared to the vehicle condition.

To assess effects of antipsychotics on arousal level, the time spent in active waking was quantified and expressed in % of total duration of the recording session during baseline condition and within discrete time points 45–60 and 105–120 min post dosing. Network connectivity between cortical regions was computed by phase coherence, which is a measure commonly used to probe the integrity of cortical neural pathways and index the functional coupling between different cortical structures at various frequencies^47^. The magnitude of EEG coherence ranges between 0 and 1: a low value indicates no similarity between the two signals, whereas values close to 1 indicate a high similarity between two-time series. Functional connectivity between the time series in different electrodes was quantified by coherence at different time points before and after administration of drugs using a generalized additive mixed model where the coherence over the frequencies was fitted using a cubic spline for the different treatment groups and the heterogeneity between the animals^48,49^. Subsequently, coherence between each pair of electrodes was concatenated every 4 s and aggregated over 15 min in each frequency bands for each animal, time point, and treatment group. Frequency bands were selected for comparison between groups based on the most prominent oscillatory and coherent activities across different brain regions. To reduce the impact of volume conduction of signals propagated from common generators, computation of all edges in which the maximum absolute value of coherence occurred at zero time lags between time series was removed.

### Drugs

All drugs were synthesized in Janssen Research and Development laboratories. For acute experiments, olanzapine (0.16, 0.64, and 2.5 mg/kg), clozapine (0.16, 0.64, and 2.5 mg/kg), and risperidone (0.16, 0.64, and 2.5 mg/kg) were formulated in H2O + 2H2T (tartaric acid) + NaCl. PCP (1.25, 2.5, and 5 mg/kg), MK801 (0.16, 0.64, and 2.5 mg/kg), and amphetamine (0.16, 0.64, and 2.5 mg/kg) were dissolved in saline solution. Solutions were subcutaneously (s.c.) administered in a volume of 5 ml/kg body weight. In the case of combined pharmacological studies, antipsychotics were administered 30 min before the drug challenge. The doses selected for PCP, MK801, and amphetamine were repeatedly found to elicit marked alterations in motor behavior and EEG patterns^50^. The doses of risperidone and olanzapine were selected based on occupancy of dopamine D2 receptor and their potency to attenuate the challenge-induced hyperlocomotor behavior. The dose of clozapine was selected based on its ability to antagonize apomorphine-induced stereotypy behavior^51^.

### Statistics

The values of consecutive 4 s epochs were averaged and drug-induced changes in EEG oscillations were calculated in blocks of 15 min for 2 h as the ratio of mean spectral power obtained following the administration of test drug vs. the mean spectral power obtained during 30 min baseline period. This procedure allows for assessment of drug-induced changes in EEG power expressed at each frequency band as a percentage of the original power, compared to the vehicle condition. Time course EEG spectral changes were submitted to a two-way multivariate ANOVA for repeated measures with two main factors (treatment and period of recordings), followed by pairwise comparisons between treatment levels for each of the 15 min periods. A one-way ANOVA was applied to compare effects of different antipsychotics on average percent time spent in active waking within discrete time point 45–60 and 105–120 min. Sample sizes including animal numbers were chosen to ensure adequate statistical power comparable to previously published papers. Data distribution of experiments was assumed to be normal and was presented as the means ± SEM. The aggregated coherences as well as the changes from baseline were analyzed per frequency band using an ANOVA with time, group, and its interaction as covariates while considering the heterogeneity between the different animals^52^. *p* values from the associated F-tests were reported as well as Least squares means from the corresponding ANOVA models. Associations between EEG parameters and activity levels were evaluated using Spearman rank-correlation analyses.

## Results

### Disruption of glutamatergic and dopaminergic transmissions affects cortical network oscillations and connectivity

**Fig. 1:**
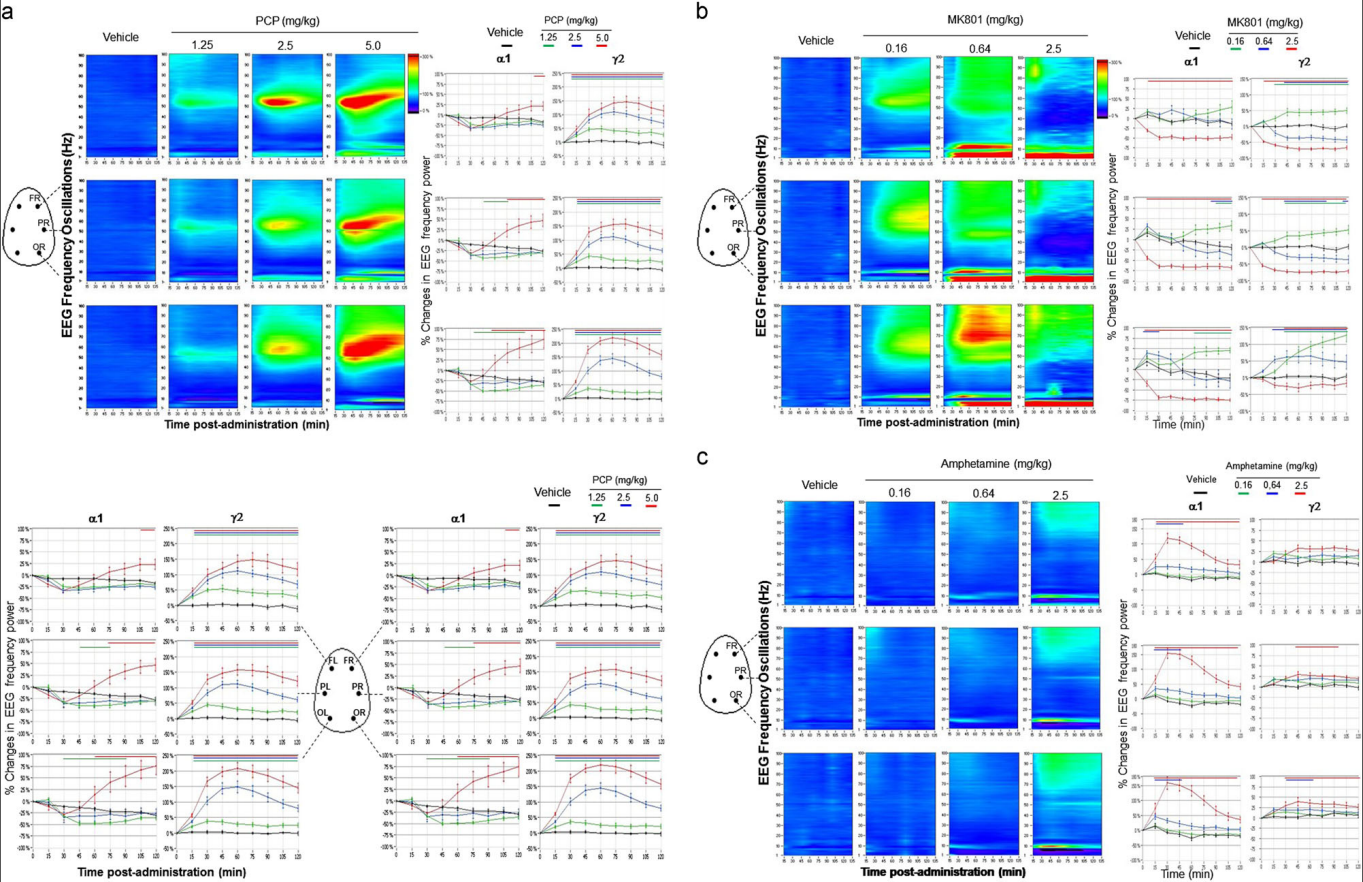
Atypical antipsychotics commonly decreased slow alpha and higher gamma network oscillations Full power spectrum expressed as a heat map in fronto–parieto–occipital cortical areas during each 15 min block of the recording session after the administration of **a** PCP (1.25, 2.5, and 5 mg/kg) in 1 one hemisphere and 2 both hemispheres for, **b** MK801 (0.16, 0.64, and 2.5 mg/kg), and **c** amphetamine (0.16, 0.64, and 2.5 mg/kg). As there was no major difference in spectral contents between hemispheres (**a**2), only right hemisphere locations along the anteroposterior axes were presented in subsequent figures (frontal right “FR”, parietal right “PR”, and occipital right “OR”). Changes in color from cold dark blue to warm red color indicates an order increase in the magnitude of oscillatory power. Curves on the right side indicate the time course changes in the oscillatory activity at alpha1 and gamma2 frequencies during 2 h after the administration of PCP, MK801, and amphetamine. Symmetrical changes in both frequency oscillations were found in both hemispheres see (**a**2) for PCP; therefore, only right hemisphere was displayed (*n* = 8 for each condition). Color-coded bars above the curves indicate intervals in which oscillatory activity difference differed from vehicle

The NMDA antagonists and the dopaminergic agonist were used to model the aberrant cortical network oscillations in rats that resemble the dysfunctional network oscillations widely described in schizophrenia. Acute administration of PCP dose-dependently enhanced aberrant network oscillatory synchrony in higher gamma frequencies, which was apparent in different brain areas (Fig. 1a1). As changes were comparable on the other brain hemisphere (see for PCP Fig. 1a2), only data from one hemisphere were presented in subsequent figures. MK801 induced a region-specific and time course changes in gamma oscillations in an inverted U-shaped relation, i.e., it increased with the lower and middle dose and decreased with the higher dose, which appeared to differ from those observed with PCP (Fig. 1b). Amphetamine at the higher dose increased higher gamma oscillations across different brain areas (Fig. 1c). Quantification of the time that oscillations was enhanced showed that elevated cortical gamma oscillations were already apparent at 15 min after the administration of drugs.

In addition to their effects on high oscillatory rhythm, both MK801 and amphetamine increased the overall slow alpha oscillatory activity in different brain regions (see heat map and specific power band in Figs. 1b, c).

### Atypical antipsychotics commonly decreased slow alpha and higher gamma network oscillations

**Fig. 2:**
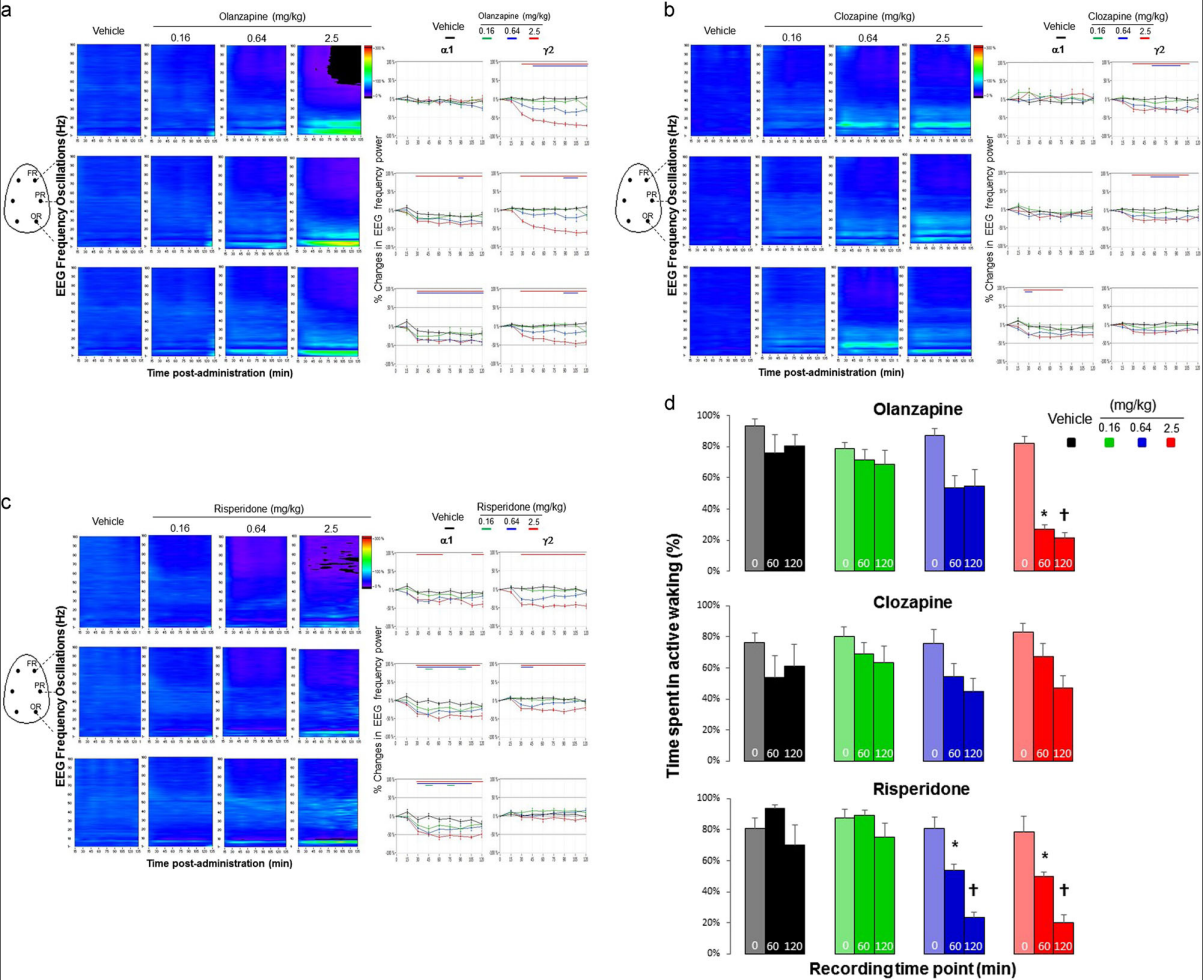
Atypical antipsychotics commonly decreased slow alpha and higher gamma network oscillations Full power spectrum expressed as a heat map in fronto–parieto–occipital cortical areas during each 15 min block of the recording session after the administration of **a** Olanzapine (0.16, 0.64, and 2.5 mg/kg), **b** Clozapine (0.16, 0.64, and 2.5 mg/kg), and **c** Risperidone (0.16, 0.64, and 2.5 mg/kg). Only right hemisphere locations along the anteroposterior axes were presented (frontal right “FR”, parietal right “PR”, and occipital right “OR”). Changes in color from cold dark blue to warm red color indicate an order increase in the magnitude of oscillatory power. Time course changes in alpha1 and gamma2 frequency activities during 2 h are shown in the right curves. Color-coded bars above the curves indicate intervals in which oscillatory activity difference differed from vehicle (*n* = 8 for each condition). **d** Time spent in epochs scored as active waking from all 4 s epochs expressed as percent change (±SEM) from the time of baseline session and within discrete time point 45–60 and 105–120 min post dosing. Note the large decrease in time spent in active waking produced by all antipsychotics at high dose. Light-coded colors indicate baseline sessions of all treatment groups (Condition 0), * and ^†^ indicate significant changes from vehicle during the time point 45–60 and 105–120 min post dosing, respectively

We present the effects of different antipsychotics on network oscillatory patterns. The hypothesis was that the antipsychotics would elicit the opposite effects on cortical network abnormalities to those described in schizophrenic patients and modeled in rats. Administration of olanzapine, clozapine, and risperidone commonly and consistently reduced oscillatory network activity in the higher gamma frequency oscillations (Figs. 2a–c, respectively). In addition, a consistent decrease in network slow alpha activity was found with the middle and higher doses of all antipsychotics, as well as an enhancement of slow theta oscillations in the parietal and occipital areas, whereas olanzapine and clozapine had additional effects on delta and fast alpha oscillations in the frontal area, respectively (Figs. 2a, b).

Antipsychotics often exhibit sedation or activity suppression. To examine the effects of different antipsychotics on the duration of arousal, we quantified the time spent in epoch scored as active waking from all 4 s epochs of the baseline session and within discrete time points 45–60 and 105–120 min post dosing. In all treatment groups, there was no major changes in time spent in baseline active waking in case of clozapine (F(3,28) = 0.23, *p* = 0.87); Olanzapine (F(3,28) = 0.48, *p* = 0.7); and risperidone (F(3,28) = 0.27, *p* = 0.84). Approximately 80% of time during baseline (Condition 0) was spent in active waking (Fig. 2d, light-color-coded bars for each treatment group). Under vehicle and at the dose of 0.16 mg/kg of different antipsychotics, animals spent up to 80% of time in active waking during the 45–60 and 105–120 min post dosing (Fig. 2d, black-colored and green-colored bars). At the dose of 0.64 mg, animals spent up to 60% of time in active waking during the 45–60 min post dosing of all antipsychotics, whereas clozapine and risperidone produced a significant decrease of epochs scored as active waking up to 45 and 20% during the 105–120 min post dosing, respectively (Fig. 2d, blue-colored bars). At the dose of 2.5 mg/kg, all antipsychotics produced a significant decrease up to 50% of epochs scored as active waking during the 105–120 min post dosing of clozapine, whereas this level reached 20% for both olanzapine and risperidone (Fig. 2d, red-colored bars).

### Antipsychotics normalized aberrant cortical network oscillations elicited by different challenges

**Fig. 3:**
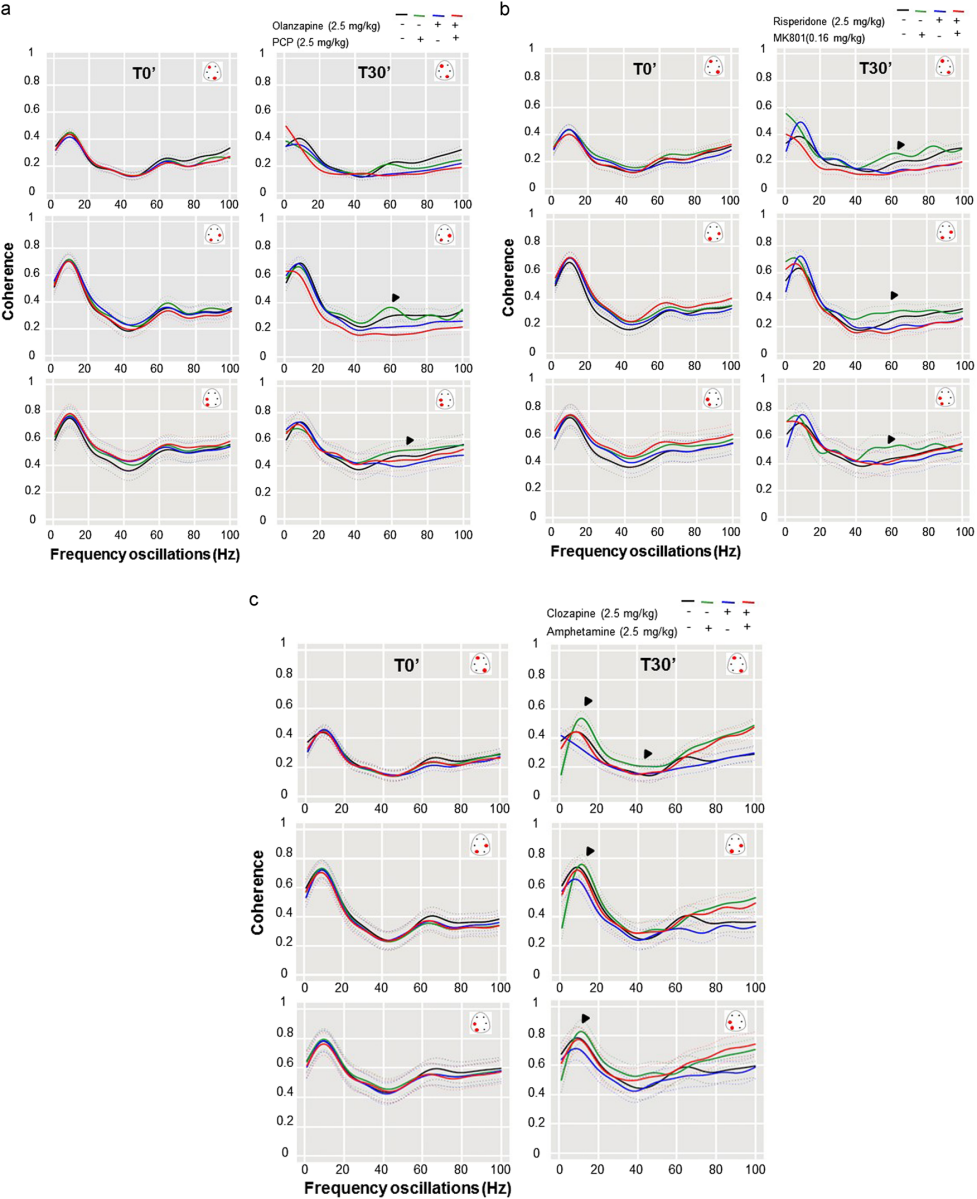
Antipsychotics normalized aberrant cortical coherent activities elicited by different challenges Coherence coefficients in recorded areas during baseline and at 30 min after the combined subcutaneous administration of **a** vehicle+vehicle, vehicle+PCP (2.5 mg/kg), olanzapine (2.5 mg/kg)+vehicle, and of olanzapine (2.5 mg/kg)+PCP (2.5 mg/kg; *n* = 8 for each condition). PCP-induced increases in coherent gamma oscillations were attenuated by olanzapine particularly in parieto-occipital areas (arrows), **b** vehicle+vehicle, risperidone (2.5 mg/kg)+vehicle, vehicle+MK801 (0.16 mg/kg), and of risperidone (2.5 mg/kg)+MK801 (0.16 mg/kg*; n* = 8 for each condition). MK801-induced increases in coherent gamma oscillations were attenuated by risperidone, **c** vehicle+vehicle, vehicle+amphetamine (2.5 mg/kg), clozapine (2.5 mg/kg)+vehicle, and of clozapine (2.5 mg/kg)+amphetamine, (*n* = 8 for each condition). Amphetamine-induced increases in coherent slow alpha oscillations was attenuated by clozapine

The potency of antipsychotics to reverse the challenge-induced aberrant network synchrony in slow alpha and higher gamma frequencies is shown in Figs. 3a–d. Pre-treatment with olanzapine (2.5 mg/kg) normalized PCP-induced abnormalities in higher gamma network oscillations and connectivity (Fig. 3a).

Risperidone normalized the pathological synchronous high gamma and slow alpha network oscillations induced in the MK801-treated rats (Fig. 3b). Similar observations were also indicated in the case of slow alpha network rhythm in MK801-treated rats.

Qualitative evaluation of the efficacy of clozapine to reverse abnormalities in EEG network oscillations and associated disorganized behavior in the amphetamine-treated rats are shown in Fig. 3c. Amphetamine-induced increases in coherent EEG network oscillations in the slow alpha oscillatory rhythm were effectively blocked by pre-treatment with clozapine, which was observed 30 min after administration of the antipsychotic.

### Correlation between higher gamma oscillations and locomotor activity

**Fig. 4:**
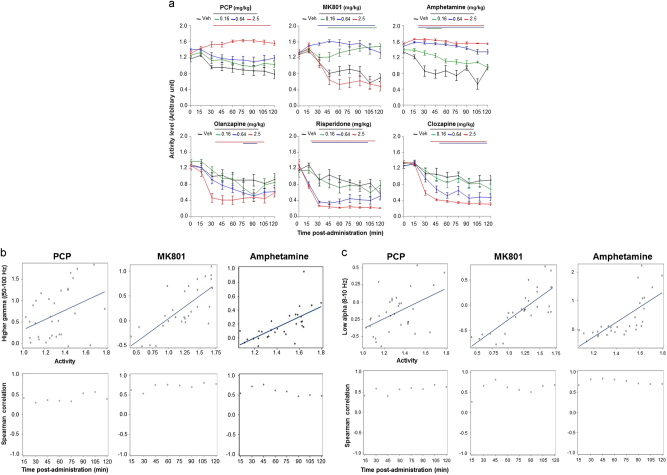
Correlation between higher gamma oscillations and locomotor activity **a** Activity levels during the first 2 h after the administration of PCP, MK801, and amphetamine (upper panels) and olanzapine, and risperidone (bottom panels). Note that all challenge drugs enhanced locomotor activity, whereas antipsychotics attenuated motion levels. **b** Spearman correlations (upper panels) at 30 min and *R*-values plotted for each time block of 15 min after the administration of different drug challenges between the higher gamma oscillations (50.2–100 Hz) and locomotor activity, and **c** the slow alpha oscillations (8–10 Hz) and locomotor activity

Analysis of motion activity at different time points showed that drug challenges enhanced, whereas different antipsychotics consistently decreased locomotor activity levels (Fig. 4, upper and bottom panels, respectively).

We have assessed whether the changes in spectral contents were correlated with changes in motion levels. Behavioral observation showed that rats treated with PCP, MK801, and amphetamine exhibited disorganized motor behavior. Time course analysis of motion levels at different time points showed that different challenge drugs were associated with a dose-dependent enhancement of activity levels, except for MK801-treated animals, which showed decreased motor levels at the higher dose (Fig. 4a, upper middle panel).

The results of a Spearman rank-correlation analysis between the higher gamma activity and motor activity at 30 min after the administration of challenge drugs are shown in Fig. 4b, upper panels. The correlation analysis showed that high gamma oscillations were positively correlated with activity levels (PCP *r* = 0.43, *p* < 0.01; MK801 *r* = 0.75, *p* < 0.0001; amphetamine *r* = 0.77, *p* < 0.0001). The scatter plot in Fig. 4b, bottom panel, shows a highly significant positive association between EEG higher gamma activity and motor activity for each time interval after the administration of all drug challenges (Spearman rank-correlation analysis for all time points in case of PCP: rS ranges between 0.38 and 0.57, all *p* values < 0.05, for MK801: rS ranges between 0.53 and 0.81, all *P* values < 0.001, and for amphetamine: rS ranges between 0.48 and 0.77, all *p* values < 0.05).

In addition, the Spearman rank-correlation analysis at 30 min showed a positive correlation between slow alpha activity (8–11 Hz) and motor activity levels (PCP *r* = 0.41, *p* < 0.02, MK801 *r* = 0.81, *p* < 0.0001 and amphetamine *r* = 0.82, *p* < 0.001), which was maintained across all time intervals of the recording session (PCP: rS ranges between 0.4 and 0.7, all *p* values < 0.01, MK801: rS ranges between 0.2 and 0.8, all *p* values < 0.001, amphetamine: rS ranges between 0.75 and 0.85, all *p* values < 0.001; Fig. 4c).

### Antipsychotics normalized challenge-induced aberrant gamma network oscillations dependently or independently from motoric behavior

**Fig. 5:**
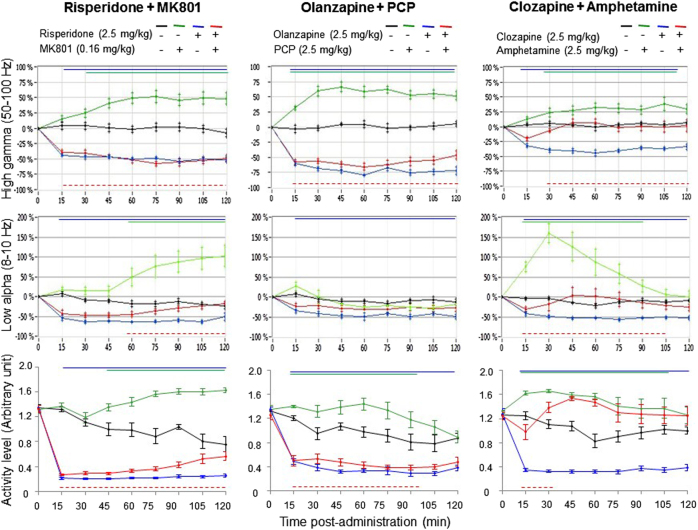
Antipsychotics normalized challenge-induced aberrant gamma network oscillations associated with pronounced motion levels Time course of concomitant changes in slow alpha-high gamma power and motion levels during each 15 min time blocks for 2 h following the combined administration of risperidone (2.5 mg/kg)+MK801 (0.16 mg/kg), olanzapine (2.5 mg/kg)+PCP (2.5 mg/kg), and clozapine (2.5 mg/kg)+amphetamine (2.5 mg/kg; *n* = 8 for each condition). Color-coded bars above curves indicate intervals in which oscillatory activity differed from vehicle, whereas curve underneath curves indicates interval in which antipsychotics consistently attenuated the effects of challenges

EEG gamma oscillatory activity is known to be modulated by motor speed, while at the same time antipsychotics are expected to reduce locomotor behavior. To rule out whether the speed of movement is the driving effect of antipsychotics, we have examined concomitant changes of EEG gamma activity and motion levels. Here, all challenge drugs significantly enhanced abnormal gamma oscillations associated with pronounced motion levels, which could last up to 120 min post administration (Fig. 5, top and bottom plots). In addition, MK801 and amphetamine increased the power in the slow alpha (8–10 Hz), starting to peak at 60 min after the administration of MK801, whereas it reached a maximum peak at 30 min after the administration of amphetamine (Fig. 5, middle left and right plots). Antipsychotics completely suppressed the concomitant abnormal oscillatory rhythms and associated hyperactive behavior in the NMDA receptor model, whereas a partial suppression of locomotor activity revealed a dissociation in the amphetamine model (Fig. 5, bottom right panel).

## Discussion

In the present studies, (1) all NMDA receptor antagonists elicited aberrant network oscillations in the higher gamma frequency range associated with pronounced hyperactivity, while a consistent abnormal slow alpha coherent oscillatory activity was observed with both amphetamine and MK801, (2) all antipsychotics decreased EEG slow alpha and higher network gamma oscillations as well as decreased motion levels, (3) pre-treatment with antipsychotics attenuated drug challenge-induced abnormal slow alpha and higher gamma network synchrony, which was mechanism-dependent.

### Relevance of aberrant network oscillations model

Network gamma synchrony selectively facilitates the communication among synchronized assemblies to ensure large numbers of cortical computations^53–55^. Schizophrenia is considered as a disconnection syndrome associated with failure in early automatic neural connectivity of important sensory inputs leading to cognitive impairments^56^. These widespread structural abnormalities are accompanied by alterations in glutamatergic and GABAergic neurotransmission and decreased expression of NMDA receptors on inhibitory interneurons, which lead to an imbalance between excitation and inhibition, hyperexcitability, and unstable coordination of cortical networks^57–59^. Aberrant synchronization in the gamma oscillatory range has been repeatedly shown to accompany many disturbed neurocognitive functions in schizophrenic patients^3,5,7,15,30,60,61^. Positive symptoms of schizophrenia are correlated with enhanced gamma oscillatory synchrony and had been associated with an attempt to adapt to a progressive loss of cortical gray matter and associated changes in cognitive and emotional function, whereas negative symptoms have been related to both enhanced and reduced high-frequency oscillations^62–64^. Therefore, network gamma oscillations may provide a plausible animal–clinical interface on studying dysfunction in the integrity of glutamatergic, dopaminergic, and GABAergic neuronal circuits in schizophrenia.

### Blockade of NMDA receptors elicited aberrant cortical network oscillations

Administration of NMDA receptor antagonists enhanced aberrant cortical network gamma oscillations.

In human and animal studies, acute and chronic administration of PCP, ketamine, and MK801 produced schizophrenia-like behavior with positive and negative symptoms associated with cognitive impairments^51,65^. The NMDA receptors have been proposed as a candidate for the synchronization of local circuits, which are prominent in the superficial cortical layers that are the main recipients of long corticocortical connections. Disinhibition of principal cells that may result from reduced interneuron excitation would facilitate the transient pathological generation of gamma oscillations. Our present results further extend previous observations showing abnormal cortical gamma oscillations associated with hyperlocomotion following systemic injection of PCP, MK801, and ketamine^40,41^. The potential mechanism underlying aberrant gamma oscillations might involve the release of monoamines such as dopamine and serotonin, which in turn increases glutamate transmission in the prefrontal cortex to elicit transient aberrant neuronal network activation^65–67^. PCP strongly binds in the hippocampus leading to increased metabolic activity and schizophrenic-like symptoms as well as disorganized behavior by activating dopaminergic neurotransmission in the nucleus accumbens, which may in turn drive the nucleus at gamma oscillatory rhythm^68^. Accordingly, muscimol-induced inactivation of the hippocampal medial septum concomitantly suppressed the occurrence of gamma oscillations and associated disorganized behavior^40^. Thus, disruption of NMDA receptor signaling may release local gamma frequency oscillations from the inhibitory control of extended networks leading to pathological increase of this rhythm.

### MK801 enhanced aberrant slow alpha network oscillations

The thalamocortical coupling generates a large network oscillatory activity in the alpha frequency range (8–11 Hz) during cortical operations. However, this rhythm may also emerge during loss of attention in the immobile state or during failure to process sensory input, and may serve to functionally disengage and reduce the processing capabilities of a given brain region. The alpha activity is decreased in engaged brain regions, whereas it increased in disengaged regions^69^. In general, theta rhythmicity has been shown to organize gamma frequency dynamics of neuronal populations during cortical computing^2,9,70,^, both of which rhythms are accompanied with reduced synchrony of alpha oscillations. Here, the enhanced alpha network activity by MK801 may silence theta and gamma activities to disengage and/or inhibit network computations by decreasing the demand of those cortical regions.

The mechanism by which MK801 elicited aberrant slow alpha oscillation associated with hyperactivity is not clear. In non-pharmacologically treated animals^71,72,^ and human^73,74^, motor activity is accompanied with changes in alpha power. The strong correlation between the magnitude of the alpha band oscillation at the onset of motion and the duration of spontaneous movement epoch is consistent with a mechanical connection to motor planning networks in parietal cortex^74–76^. Pharmacological evidence has shown a functional interaction between glutamatergic–serotoninergic–dopaminergic systems and behaviors related to NMDA receptor. On the one hand, inhibition of NMDA receptors has been shown to reduce the inhibitory GABAergic signaling from PV-containing interneurons and to consequently increase the rate of pyramidal neurons and glutamate release^77,78^ as well as psychotomimetic activity^79^. On the other hand, a variety of antagonists having selectivity at 5-HT2a and D2 receptors attenuated the disruptive effect of NMDA blockade on locomotor activity^80–84^.

In the present study, MK801-induced aberrant slow network alpha frequency oscillation associated with hyperactivity was attenuated by risperidone, possibly due to its atypical character ascribed to combined antagonism at 5-HT2a/D2 receptors. Thus, these results further extend the evidence for functional interactions between NMDA and 5-HT2a/D2 receptor systems and suggest that abnormal EEG power and behavioral disinhibition actions of MK801 may largely be attributed to pronounced enhancement of glutamate neurotransmission following the activation of presynaptic and postsynaptic serotonin and/or dopaminergic receptors^66,81,^.

### Activation of dopaminergic receptors affected slow alpha and higher gamma network oscillatory rhythms

Administration of amphetamine elicited robust coherent EEG slow alpha network activity at the dose that induced stereotypic behavior.

Amphetamine is widely used to model positive symptoms of schizophrenia such as delusions, hallucinations, and disorganized behavior by increasing dopaminergic function in the brain, particularly in the nucleus accumbens^35^. Amphetamine is known to produce active arousal stereotypic behavior, i.e., sniffing and licking, which could be suppressed by intra-accumbens injection of either dopamine antagonist^85^, or glutamate receptor blockade^86^. There is scarce evidence for a direct influence of the dopaminergic transmission on neuronal oscillations in schizophrenia. Administration of psychomotor stimulant amphetamine or the dopamine agonist apomorphine causes a prolonged pathological increase of cortical gamma oscillations likely through increased excitability of fast-spiking GABAergic interneurons^87,88^.

In the present work, amphetamine consistently induced coherent alpha and gamma activities associated with signs of disorganized behavior. The enhancement of higher gamma oscillations may result from its influence on fast-spiking PV-containing interneurons, while the particular enhancement of slow alpha oscillations might derive from the stimulation of presynaptic dopaminergic D1 receptors and postsynaptic dopaminergic D2 receptors^89^. In a similar vein, amphetamine may enhance 5-HT neurotransmission in a similar way as hallucinogenic LSD or DOM, which have been shown to attenuate 5-HT release through activation of presynaptic 5-HT2a/2c leading to increased glutamate release in the prefrontal cortex, stereotypic behavior, and increased the power of the alpha1 rhythm^66,90^.

### Clinically effective antipsychotics attenuate aberrant network oscillations

Clozapine, olanzapine, and risperidone had highly distinctive EEG fingerprints and caused similar attenuation of the NMDA antagonists and amphetamine-induced aberrant network oscillations in different cortical regions.

Increased gamma oscillations have consistently been observed in schizophrenic patients^30,91,92^. Interestingly, the effectiveness of antipsychotic drugs in relieving schizophrenic symptoms was correlated with the decrease in fast cortical activity^93,94^. In addition, both typical and atypical antipsychotics demonstrated a potency to decrease basal gamma and theta power; however, it is not clear whether this medication provides benefit for cognitive function in schizophrenia^35,95,96^. Moreover, the typical antipsychotic haloperidol at a therapeutic dose that occupies >70% of D2 receptors significantly reduced PCP, MK801, and ketamine-induced higher gamma oscillatory rhythm and hyperlocomotion^97,98^. Likewise, all effective antipsychotics inhibit dopaminergic neurotransmission and exhibit different affinities for 5-HT receptors (5-HT1;2;3;6;7), and serotonin transporter^96–98^. The increased coherent slow alpha rhythm elicited by amphetamine was likely mediated through presynaptic D1 and/or postsynaptic D2 dopaminergic.

As the atypical antipsychotics display combined antagonist activity at D2 and 5-HT2a/c receptors, their efficacy to increase 5-HT/DA release would decrease the slow alpha network synchrony, an effect that may contribute to antisychotic therapeutic actions^99^. The current study extends on a previous report showing a late decrease of striatal alpha1 spectral power by atypical antipsychotic drugs in rats^100^. The fact that antipsychotics attenuated the NMDA receptor antagonist-induced aberrant oscillations associated with hyperactivity may suggest an indirect interaction with the glutamatergic system rather than the dopaminergic system. Assessment of specific pharmacological ligands into D1/2 receptor function would be an obvious way to examine this hypothesis and possible relationship to sedative effects of antipsychotics.

### Attenuation of aberrant cortical alpha/gamma oscillations and related hyperactive behavior is mechanism-dependent

Gamma power in the rodent prefrontal cortex increases with running speed, and gamma power has been shown to be higher in fast-moving rats under saline and in rats treated with an NMDA antagonist^40,101^. Alpha power has been associated with stereotypic behavior elicited by different hallucinogenic and psychostimulant drugs^90^. This raises the question as to whether the effects of antipsychotics on EEG power are a result of the reduced locomotion^102^, rather than a direct effect on cortical processing.

In the present work, PCP had no consistent effect on theta frequency rhythm critically involved in the moment-to-moment voluntary movement indicating that this rhythm is not a key activity underlying the hyperactive behavior in the PCP model. However, both MK801 and amphetamine increased the power in the 8–10 Hz known to be linked to abnormal behavior elicited by hallucinogenic and psychostimulant drugs^90^. Both risperidone and olanzapine attenuated concomitantly evoked gamma network oscillations and hyperlocomotor behavior, as well as additional alpha rhythm in case of MK801. However, clozapine decreased amphetamine-induced gamma oscillatory rhythm appeared to last longer and dissociated from behavioral hyperactivity (Figure 5, right panel). Remarkably, attenuation of alpha rhythm in the amphetamine model, known to accompany stereotypic behavior such as circling and head weaving, was short-lived, peaking at 15–30 min followed by an active exploring behavior for the remaining recording session.

Gamma oscillatory rhythm has been suggested to influence behavioral response via the nucleus accumbens by interfering with glutamate or dopamine release^40^. Accordingly, the administration of the GABAA agonist muscimol in the medial septum suppressed completely locomotor activity in animals treated with PCP and only partially in amphetamine-treated animals^40,103^. Consistent with this view, antipsychotics completely suppressed the hyperactivity that accompanied the increase in the gamma rhythm elicited by NMDA receptor blockade but not in dopaminergic receptor activation. These findings undermine the argument that attenuation of aberrant network oscillations may underlie the antipsychotic activity, which may be mechanistically associated or dissociated from having an active role in the hyperactive behavior in the NMDA receptor model or the dopaminergic receptor model, respectively.

### Implications

Convergent clinical literature indicates abnormalities in synchronization of EEG gamma oscillation as an endophenotype in schizophrenic patients. In rodents, acute NMDA receptor blockade produced schizophrenia-like behavior and cognitive deficits associated with increased cortical network excitability and impaired gamma oscillation synchrony. Synchronization of gamma oscillations has a wider relevance in cortical networks for behavioral and cognitive phenomena, and emerging views suggest that development of medications that could improve cognition is a major step forward in achieving better functional outcome in schizophrenic patients. Targeting gamma-frequency deficits and the circuit insults, the index could have implications to improve perceptual abnormalities in a broad group of schizophrenia patients^104^. Novel therapeutic strategies that could normalize aberrant high gamma network oscillations and/or elevate gamma power during cognitive paradigms may be promising candidates for restoring gamma abnormalities in schizophrenia and associated cognitive impairments. Therefore, the ability to induce and maintain network oscillations within the gamma range in the rat offers a promising translational tool in preclinical research that may enhance the chance of screening and identifying novel drugs with antipsychotic potential with cognitive enhancing properties.

## References

[1] Buzsáki G, Draguhn A (2004). Neuronal oscillations in cortical networks. Science.

[2] Colgin LL (2009). Frequency of gamma oscillations routes flow of information in the hippocampus. Nature.

[3] Debener S, Herrmann CS, Kranczioch C, Gembris D, Engel AK (2003). Top-down attentional processing enhances auditory evoked gamma band activity. Neuroreport.

[4] Fell J (2001). Human memory formation is accompanied by rhinal-hippocampal coupling and decoupling. Nat. Neurosci..

[5] Ford JM, Gray M, Faustman WO, Heinks TH, Mathalon DH (2005). Reduced gamma-band coherence to distorted feedback during speech when what you say is not what you hear. Int. J. Psychophysiol..

[6] Fries P (2009). Neuronal gamma-band synchronization as a fundamental process in cortical computation. Annu. Rev. Neurosci..

[7] Gruber T, Tsivilis D, Montaldi D, Müller MM (2004). Induced gamma band responses: an early marker of memory encoding and retrieval. Neuroreport.

[8] Lutzenberger W, Ripper B, Busse L, Birbaumer N, Kaiser J (2002). Dynamics of gamma-band activity during an audiospatial working memory task in humans. J. Neurosci..

[9] Palva S (2002). Distinct gamma-band evoked responses to speech and non-speech sounds in humans. J. Neurosci..

[10] Tallon-Baudry C, Bertrand O, Hénaff MA, Isnard J, Fischer C (2005). Attention modulates gamma-band oscillations differently in the human lateral occipital cortex and fusiform gyrus. Cereb. Cortex.

[11] Gray CM, Konig P, Engel AK, Singer W (1989). Oscillatory responses in cat visual cortex exhibit inter-columnar synchronization which reflects global stimulus properties. Nature.

[12] Lesh TA, Niendam TA, Minzenberg MJ, Carter CS (2011). Cognitive control deficits in schizophrenia: mechanisms and meaning. Neuropsychopharmacology.

[13] Roux F, Uhlhaas PJ (2014). Working memory and neural oscillations: α-γ versus θ-γ codes for distinct WM information?. Trends Cogn. Sci..

[14] Lee KH, Williams LM, Breakspear M, Gordon E (2003). Synchronous gamma activity: a review and contribution to an integrative neuroscience model of schizophrenia. Brain Res. Brain Res. Rev..

[15] Phillips WA, Silverstein SM (2003). Convergence of biological and psychological perspectives on cognitive coordination in schizophrenia. Behav. Brain Sci..

[16] Bartos M, Vida I, Jonas P (2007). Synaptic mechanisms of synchronized gamma oscillations in inhibitory interneuron networks. Nat. Rev. Neurosci..

[17] Fries P, Neuenschwander S, Engel AK, Goebel R, Singer W (2001). Modulation of oscillatory neuronal synchronization by selective visual attention. Science.

[18] Lewis DA, Volk DW, Hashimoto T (2004). Selective alterations in prefrontal cortical GABA neurotransmission in schizophrenia: a novel target for the treatment of working memory dysfunction. Psychopharmacology.

[19] Kwon JS (1999). Gamma frequency-range abnormalities to auditory stimulation in schizophrenia. Arch. Gen. Psychiatry.

[20] Spencer KM (2003). Abnormal neural synchrony in schizophrenia. J. Neurosci..

[21] Whittington MA, Cunningham MO, LeBeau FE, Racca C, Traub RD (2011). Multiple origins of the cortical γ rhythm. Dev. Neurobiol..

[22] Addington AM (2005). GAD1 (2q31.1), which encodes glutamic acid decarboxylase (GAD67), is associated with childhood-onset schizophrenia. Mol. Psychiatry.

[23] Sohal VS, Zhang F, Yizhar O, Deisseroth K (2009). Parvalbumin neurons and gamma rhythms enhance cortical circuit performance. Nature.

[24] Whittington MA, Traub RD (2003). Interneuron diversity series: inhibitory interneurons and network oscillations in vitro. Trends Neurosci..

[25] Benes FM (2010). Amygdalocortical circuitry in schizophrenia: from circuits to molecules. Neuropsychopharmacology.

[26] Benes FM, Berretta S (2001). GABAergic interneurons: implications for understanding schizophrenia and bipolar disorder. Neuropsychopharmacology.

[27] Brisch R (2014). The role of dopamine in schizophrenia from a neurobiological and evolutionary perspective: old fashioned, but still in vogue. Front. Psychiatry.

[28] Coyle JT (2004). The GABA-glutamate connection in schizophrenia: which is the proximate cause?. Biochem. Pharmacol..

[29] Perez SM, Lodge DJ (2014). New approaches to the management of schizophrenia: focus on aberrant hippocampal drive of dopamine pathways. Drug. Des. Devel. Ther..

[30] Uhlhaas PJ, Singer W (2010). Abnormal neural oscillations and synchrony in schizophrenia. Nat. Rev. Neurosci..

[31] Bondi C, Matthews M, Moghaddam B (2012). Glutamatergic animal models of schizophrenia. Curr. Pharm. Des..

[32] Jackson ME, Homayoun H, Moghaddam B (2004). NMDA receptor hypofunction produces concomitant firing rate potentiation and burst activity reduction in the prefrontal cortex. Proc. Natl. Acad. Sci. USA.

[33] Javitt DC, Jayachandra M, Lindsley RW, Specht CM, Schroeder CE (2000). Schizophrenia-like deficits in auditory P1 and N1 refractoriness induced by the psychomimetic agent phencyclidine (PCP). Clin. Neurophysiol..

[34] Javitt DC, Zukin SR (1991). Recent advances in the phencyclidine model of schizophrenia. Am. J. Psychiatry.

[35] Jones CA, Watson DJ, Fone KC (2011). Animal models of schizophrenia. Br. J. Pharmacol..

[36] Meltzer HY (2013). Translating the N-methyl-D-aspartate receptor antagonist model of schizophrenia to treatments for cognitive impairment in schizophrenia. Int. J. Neuropsychopharmacol..

[37] Behrendt RP, Young C (2004). Hallucinations in schizophrenia, sensory impairment, and brain disease: a unifying model. Behav. Brain Sci..

[38] Hakami T (2009). NMDA receptor hypofunction leads to generalized and persistent aberrant gamma oscillations independent of hyperlocomotion and the state of consciousness. PLoS. ONE.

[39] Light GA (2006). Gamma band oscillations reveal neural network cortical coherence dysfunction in schizophrenia patients. Biol. Psychiatry.

[40] Ma J, Leung LS (2000). Relation between hippocampal gamma waves and behavioral disturbances induced by phencyclidine and methamphetamine. Behav. Brain Res..

[41] Pinault D (2008). N-methyl d-aspartate receptor antagonists ketamine and MK-801 induce wake-related aberrant gamma oscillations in the rat neocortex. Biol. Psychiatry.

[42] Spencer KM (2008). Visual gamma oscillations in schizophrenia: implications for understanding neural circuitry abnormalities. Clin. EEG Neurosci..

[43] Boutros NN, Korzyukuv O, Jansen B, Feingold A, Bell M (2004). Sensory gating deficits during the mid-latency phase of information processing in medicated schizophrenia patients. Psychiatry Res..

[44] Haig AR (2000). Gamma activity in schizophrenia: evidence of impaired network binding?. Clin. Neurophysiol..

[45] Veiga H (2003). Neurocortical electrical activity tomography in chronic schizophrenics. Arq. Neuropsiquiatr..

[46] Ahnaou A, Huysmans H, Jacobs T (2014). Drinkenburg WHIM. Cortical EEG oscillations and networks connectivity as efficacy indices for assessing drugs with cognition enhancing potential. Neuropharmacology.

[47] Thatcher RW (2012). Coherence, phase differences, phase shift, and phase lock in EEG/ERP analyses. Dev. Neuropsychol..

[48] Chatfield, C. *The Analysis of Time Series : An Introduction* (Chapman and Hall, London, 1975).

[49] Priestley M. B. *Spectral Analysis and Time Serie*. Vol. 1 and 2 (Elsevier Academic Press, London, 1981).

[50] Ahnaou A, Megens AA, Drinkenburg WH (2003). The atypical antipsychotics risperidone, clozapine and olanzapine differ regarding their sedative potency in rats. Neuropsychobiology.

[51] Ahnaou A, Biermans R, Drinkenburg WH (2016). Modulation of mGlu2 receptors, but not PDE10A inhibition normalizes pharmacologically-induced deviance in auditory evoked potentials and oscillations in conscious rats. PLoS. ONE.

[52] Verbeke G. and Molenberghs G. *Linear mixed models for longitudinal data,' Springer Series in Statistics* (Springer, 2000).

[53] Azouz R, Gray CM (2003). Adaptive coincidence detection and dynamic gain control in visual cortical neurons in vivo. Neuron.

[54] Salinas E, Sejnowski TJ (2000). Impact of correlated synaptic input on output firing rate and variability in simple neuronal models. J. Neurosci..

[55] Sejnowski TJ, Paulsen O (2006). Network oscillations: emerging computational principles. J. Neurosci..

[56] Insel TR (2010). Rethinking schizophrenia. Nature.

[57] Bitanihirwe BKY, Lim MP, Kelley JF, Kaneko T, Woo TUW (2009). Glutamatergic deficits and parvalbumin-containing inhibitory neurons in the prefrontal cortex in schizophrenia. BMC Psychiatry.

[58] Rubenstein JL, Merzenich MM (2003). Model of autism: increased ratio of excitation/inhibition in key neural systems. Genes. Brain Behav..

[59] Woo TU, Walsh JP, Benes FM (2004). Density of glutamic acid decarboxylase 67 messenger RNA-containing neurons that express the N-methyl-D-aspartate receptor subunit NR2A in the anterior cingulate cortex in schizophrenia and bipolar disorder. Arch. Gen. Psychiatry.

[60] Barr MS (2010). Evidence for excessive frontal evoked gamma oscillatory activity in schizophrenia during working memory. Schizophr. Res..

[61] Herrmann CS, Demiralp T (2005). Human EEG gamma oscillations in neuropsychiatric disorders. Clin. Neurophysiol..

[62] Mulert C, Kirsch V, Pascual-Marqui R, McCarley RW, Spencer KM (2011). Long-range synchrony of γ oscillations and auditory hallucination symptoms in schizophrenia. Int. J. Psychophysiol..

[63] Spencer KM (2004). Neural synchrony indexes disordered perception and cognition in schizophrenia. Proc. Natl. Acad. Sci. USA.

[64] Williams LM (2009). Neural synchrony in patients with a first episode of schizophrenia: tracking relations with grey matter and symptom profile. J. Psychiatry Neurosci..

[65] Bubeníková-Valesová V, Horácek J, Vrajová M, Höschl C (2008). Models of schizophrenia in humans and animals based on inhibition of NMDA receptors. Neurosci. Biobehav. Rev..

[66] Aghajanian GK, Marek GJ (2000). Serotonin model of schizophrenia: emerging role of glutamate mechanisms. Brain Res. Brain Res. Rev..

[67] Takahat R, Moghaddam B (1998). Glutamatergic regulation of basal and stimulus-activated dopamine release in the prefrontal cortex. J. Neurochem..

[68] Sebban C, Tesolin-Decros B, Ciprian-Ollivier J, Perret L, Spedding M (2002). Effects of phencyclidine (PCP) and MK 801 on the EEGq in the prefrontal cortex of conscious rats; antagonism by clozapine, and antagonists of AMPA-, alpha(1)- and 5-HT(2A)-receptors. Br. J. Pharmacol..

[69] Klimesch W, Sauseng P, Hanslmavr S (2007). EEG alpha oscillations: the inhibition-timing hypothesis. Brain Res. Rev..

[70] Sirota A (2008). Entrainment of neocortical neurons and gamma oscillations by the hippocampal theta rhythm. Neuron.

[71] Anderson RW, Strowbridge BW (2014). α-Band oscillations in intracellular membrane potentials of dentate gyrus neurons in awake rodents. Learn. Mem..

[72] Nerad L, Bilkey DK (2005). Ten- to 12-Hz EEG oscillation in the rat hippocampus and rhinal cortex that is modulated by environmental familiarity. J. Neurophysiol..

[73] Capotosto P, Babiloni C, Romani GL, Corbetta M (2009). Frontoparietal cortex controls spatial attention through modulation of anticipatory alpha rhythms. J. Neurosci..

[74] Kerr CE (2011). Effects of mindfulness meditation training on anticipatory alpha modulation in primary somatosensory cortex. Brain Res. Bull..

[75] Nilsson M, Waters S, Waters N, Carlsson A, Carlsson ML (2001). A behavioural pattern analysis of hypoglutamatergic mice-effects of four different antipsychotic agents. J. Neural Transm..

[76] Pfurtscheller G, Aranibar A (1979). Evaluation of event-related desynchronization (ERD) preceding and following voluntary self-paced movement. Electroencephalogr. Clin. Neurophysiol..

[77] Homayoun H, Moghaddam B (2007). NMDA receptor hypofunction produces opposite effects on prefrontal cortex interneurons and pyramidal neurons. J. Neurosci..

[78] Xi D, Zhang W, Wang HX, Stradtman GG, Gao WJ (2009). Dizocilpine (MK-801) induces distinct changes of N-methyl-D-aspartic acid receptor subunits in parvalbumin-containing interneurons in young adult rat prefrontal cortex. Int. J. Neuropsychopharmacol..

[79] Razoux F, Garcia R, Léna I (2007). Ketamine, at a dose that disrupts motor behavior and latent inhibition, enhances prefrontal cortex synaptic efficacy and glutamate release in the nucleus accumbens. Neuropsychopharmacology.

[80] Iravani MM, Muscat R, Kruk ZL (1999). MK-801 interaction with the 5-HT transporter: a real-time study in brain slices using fast cyclic voltammetry. Synapse.

[81] Maroteaux L (2017). New therapeutic opportunities for 5-HT2 receptor ligands. Pharmacol. Ther..

[82] Maurel-Remy S, Bervoets K, Millan MJ (1995). Blockade of phencyclidine-induced hyperlocomotion by clozapine and MDL 100,907 in rats reflects antagonism of 5-HT2A receptors. Eur. J. Pharmacol..

[83] Ninan I, Kulkarni SK (1998). 5-HT2A receptor antagonists block MK-801-induced stereotypy and hyperlocomotion. Eur. J. Pharmacol..

[84] Krebs-Thomson K, Lehmann-Masten V, Naiem S, Paulus MP, Geyer MA (1998). Modulation of phencyclidine-induced changes in locomotor activity and patterns in rats by serotonin. Eur. J. Pharmacol..

[85] Boye SM, Grant RJ, Clarke PB (2001). Disruption of dopaminergic neurotransmission in nucleus accumbens core inhibits the locomotor stimulant effects of nicotine and D-amphetamine in rats. Neuropharmacology.

[86] Stratford TR, Swanson CJ, Kelley A (1998). Specific changes in food intake elicited by blockade or activation of glutamate receptors in the nucleus accumbens shell. Behav. Brain Res..

[87] Berke JD (2009). Fast oscillations in cortical-striatal networks switch frequency following rewarding events and stimulant drugs. Eur. J. Neurosci..

[88] Brown P (2001). Dopamine dependency of oscillations between subthalamic nucleus and pallidum in Parkinson's disease. J. Neurosci..

[89] Stahl D, Ferger B, Kuschinsky K (1997). Sensitization to d-amphetamine after its repeated administration: evidence in EEG and behaviour. Naunyn. Schmiede. Arch. Pharmacol..

[90] Dimpfel W, Spüler M, Nichols DE (1989). Hallucinogenic and stimulatory amphetamine derivatives: fingerprinting DOM, DOI, DOB, MDMA, and MBDB by spectral analysis of brain field potentials in the freely moving rat (Tele-Stereo-EEG). Psychopharmacology.

[91] Haenschel C, Linden D (2011). xploring intermediate phenotypes with EEG: working memory dysfunction in schizophrenia. Behav. Brain Res..

[92] Turetsky BI (2007). Neurophysiological endophenotypes of schizophrenia: the viability of selected candidate measures. Schizophr. Bull..

[93] Itil TM (1982). The use of electroencephalography in the practice of psychiatry. Psychosomatics.

[94] Saletu B, Saletu M, Itil TM, Marasa J (1972). The relationship between somatosensory evoked potential and quantitatively analysed EEG during psychotropic drug treatment. Psychophysiology.

[95] Kikuchi M (2007). T. Native EEG and treatment effects in neuroleptic-naïve schizophrenic patients: time and frequency domain approaches. Schizophr. Res..

[96] Minzenberg MJ (2010). Gamma oscillatory power is impaired during cognitive control independent of medication status in first-episode schizophrenia. Neuropsychopharmacology.

[97] Horácek J (2000). Novel antipsychotics and extrapyramidal side effects. Theory and reality. Pharmacopsychiatry.

[98] Kapur S, Seeman P (2001). Does fast dissociation from the dopamine D(2) receptor explain the action of atypical antipsychotics? A new hypothesis. Am. J. Psychiatry.

[99] Marek GJ, Carpenter LL, McDougle CJ, Price LH (2003). Synergistic action of 5-HT2A antagonists and selective serotonin reuptake inhibitors in neuropsychiatric disorders. Nuropsychopharmacology.

[100] Dimpfel W (2007). Characterization of atypical antipsychotic drugs by a late decrease of striatal alpha1 spectral power in the electropharmacogram of freely moving rats. Br. J. Pharmacol..

[101] Molina LA, Skelin I, Gruber AJ (2014). Acute NMDA receptor antagonism disrupts synchronization of action potential firing in rat prefrontal cortex. PLoS. ONE.

[102] Bardgett ME, Baum KT, O'Connell SM, Lee NM, Hon JC (2006). Effects of risperidone on locomotor activity and spatial memory in rats with hippocampal damage. Neuropharmacology.

[103] Ma J, Leung LW (1999). Medial septum mediates the increase in post-ictal behaviors and hippocampal gamma waves after an electrically induced seizure. Brain Res..

[104] Whittington MA, Faulkner HJ, Doheny HC, Traub RD (2000). Neuronal fast oscillations as a target site for psychoactive drugs. Pharmacol. Ther..

